# Facile preparation of a cost-effective platform based on ZnFe_2_O_4_ nanomaterials for electrochemical cell detection

**DOI:** 10.1038/s41598-023-31377-2

**Published:** 2023-03-27

**Authors:** Fereshteh Vajhadin, Mohammad Mazloum-Ardakani, Mahdie Hemati, Seyed Mohammad Moshtaghioun

**Affiliations:** 1 Department of Chemistry, Faculty of Science, Yazd University, Yazd, 8915818411 Iran; 2 Department of Clinical Biochemistry, Faculty of Medicine, Shahid Sadoughi University of Medical Sciences, Yazd, Iran; 3 Medical Nanotechnology & Tissue Engineering Research Center, Yazd Reproductive Sciences Institute, Shahid Sadoughi University of Medical Sciences, Yazd, Iran; 4 Department of Biology, Faculty of Science, Yazd University, Yazd, Iran

**Keywords:** Biochemistry, Chemistry

## Abstract

Circulating tumor cells (CTCs) are important tumor markers that indicate early metastasis, tumor recurrence, and treatment efficacy. To identify and separate these cells from the blood, new nanomaterials need to be developed. The present study explored the potential application of ZnFe_2_O_4_ magnetic nanoparticles in capturing CTCs with cell surface markers. Folic acid was coupled to l-cysteine-capped ZnFe_2_O_4_ nanoparticles (ZC) to provide binding sites on ZnFe_2_O_4_ nanoparticles for the recognition of folate bioreceptors, which are highly expressed in MCF-7 breast cancer cells. The cytotoxicity of ZnFe_2_O_4_ nanoparticles and ZC against MCF-7 was analyzed with the MTT assay. After 24 h of incubation, there were IC50 values of 702.6 and 805.5 µg/mL for ZnFe_2_O_4_ and ZC, respectively. However, after 48 h of incubation, IC50 values of ZnFe_2_O_4_ and ZC were reduced to 267.3 and 389.7 µg/mL, respectively. The cell quantification was conducted with magnetically collected cells placed on a glassy carbon electrode, and the differential pulse voltammetry (DPV) responses were analyzed. This cost-effective ZnFe_2_O_4_-based biosensing platform allowed cancer cell detection with a limit of detection of 3 cells/mL, ranging from 25 to 10^4^ cells/mL. In future, these functionalized zinc ferrites may be used in electrochemical cell detection and targeted cancer therapy.

## Introduction

Breast cancer is a common malignancy that threats the lives of many women^1^. The commonly used clinical methods for breast cancer diagnosis include mammography, magnetic positron emission tomography (PET), computerized tomography (CT), and resonance imaging (MRI)^2^. The traditional cancer diagnostic approaches often require high-cost instruments and time-consuming procedures. In addition, they might be insensitive enough to show indeterminate results, thereby causing a delay in cancer diagnosis. As an example, MRI suffers from low resolution, which restricts accurate tumor detection^3^. Therefore, developing certain platforms through simple, inexpensive, and sensitive approaches is of great importance to fight cancer.

The main reason for cancer treatment failure is metastasis, which is mainly caused by the spread of tumor cells in the body fluids^4^. Tumor cells that separate from a tumor tissue and travel in the blood are referred to as circulating tumor cells (CTCs). The number of CTCs in the blood of cancer patients is estimated to be about 10–100 in 1 mL of blood^5,6^. The number of CTCs in blood correlates to the metastatic risk, disease-free status, and tumor recurrence^7^. At present, a widely-used CTC assay is CellSearch, which has been approved by the U.S. Food and Drug Administration (FDA). This assay benefits from magnetic cell separation by Fe_3_O_4_ nanomaterials^5^. CellSearch is a complex, high-cost, and time-consuming procedure. Although it can separate the epithelial cell adhesion molecule (EpCAM) positive CTCs, it cannot isolate EpCAM negative CTCs^8,9^. Considering the small number of CTCs in the peripheral blood and CTCs’ heterogeneity, there is a need for other systems to capture a wider spectrum of CTCs and assist in the clinical management of cancer patients.

Spinel-type metal oxides with the general formula of (AFe_2_O_4_, A = Co, Ni, Cd, and Zn) are excellent candidates for the magnetic separation of cells. This is owing to their numerous advantages such as tunable composition and structure, enhanced chemical stability, and the magnetic properties that allow their capturing by applying magnetic fields. These magnetic metal oxides can be highly beneficial in cancer therapy^10^, wound healing^11,12^, sensors^13,14^, catalysts^15^, supercapacitors^16^, and batteries^17^. In our previous studies, we demonstrated the potential application of functionalized CoFe_2_O_4_ nanomaterials in cytosensing^18,19^. However, the presence of toxic Co^2+^ ions in these spinel ferrites may limit their applicability. Hence, this study was designed to examine the potential capability of functionalized ZnFe_2_O_4_ with superior cell biocompatibility in electrochemical cell biosensing.

ZnFe_2_O_4_ possess excellent properties such as low toxicity, cost-effectiveness, and good magnetic properties for the potential applications in sensing platforms^13,20,21^. The synthesis of ZnFe_2_O_4_ nanomaterials is very feasible and their precursors are abundant, allowing mass production^22^. The outstanding catalytic activities of ZnFe_2_O_4_ nanomaterials, which are attributed to the synergetic interactions between Zn and Fe, make them a great candidate for the construction of non-enzymatic electrochemical sensing platforms^23^. A combination of ZnFe_2_O_4_ nanoparticles, Pd, and reduced graphene oxide that modified a glassy carbon electrode was introduced for the non-enzymatic detection of H_2_O_2_^24^. With advantages such as high chemical stability, magnetic properties, and electrocatalytic activities, ZnFe_2_O_4_ nanoparticles can be easily functionalized by targeting ligands for selective cell isolation and non-enzymatic electrochemical cell sensing.

The modification of ZnFe2O4 nanoparticles is more beneficial when using non-toxic biomaterials than with chemical materials that might adversely affect cell biocompatibility. Accordingly, l-cysteine, an inexpensive amino acid with zero toxicity, is selected for the modification of the ZnFe_2_O_4_ surface. This material not only offers a favorable medium for cell immobilization^25^ but also has a great metal chelating ability to enhance metal surface coating^26^. Moreover, l-cysteine provides a large number of functional groups for binding to affinity-based moieties such as folic acid (FA)^27^. In addition to giving l-cysteine antifouling properties, embedding PEG on the surface enhances non-surface fouling properties through a hydrophilic layer that attracts water molecules, suppressing non-specific binding of proteins and other molecules^28,29^. Folic acid is a targeting ligand with robust interactions with folate bioreceptors, which are highly expressed on the surface of various epithelial cancer cells as in lung, colorectal, ovarian, endometrial, colorectal, and renal cell carcinomas^30,31^. The high expression of folate bioreceptors is associated with advanced cancer stages, which acts as a negative prognostic factor^32^. Thus, FA-functionalized magnetic nanomaterials enable to capture a wide range of cancer cells.

So far, many techniques including quartz crystal microbalance (QCM)^33^, surface plasmon resonance^34^, fluorescence^35^, and electrochemical platforms^36,37^ have been used for CTC detection. Among them, electrochemical platforms are especially attractive for cell biosensing due to such advantages as ease of operation, cost-effectiveness, high sensitivity, and portability^38^. The challenge in the fabrication of an electrochemical platform for CTC quantification is the preparation of the electrode surface for the biosensing of CTCs. Various factors including the orientation of immobilized recognition elements on the electrode surface, their density, the time of cell incubation, and the type and amount of the electrode modifier alter the sensitivity of such platforms. The time-consuming and multi-step processes of electrode modification lead to the difficulty of cytosensing and the reduction of their repeatability^37–39^. These processes also suffer from operator variance. A potential solution is the direct electrochemical detection of magnetically captured cancer cells on the electrode. It reduces the use of chemical reagents and offers more eco-friendly and cost-effective ways of cytosensing^40^.

The present study makes use of functionalized ZnFe_2_O_4_ nanoparticles to introduce a straightforward, efficient and direct electrochemical cell-based assay. To this end, ZnFe_2_O_4_ nanoparticles are synthesized with in one-step route by a simple hydrothermal process without any surfactant. Then, the surface of the ZnFe_2_O_4_ nanoparticles is functionalized with l-cysteine, PEG and FA, namely ZC, for the cell-friendly magnetic separation and the electrochemical quantification of the captured cells. After the cells are captured with ZC under the effect of external magnetic fields, they drop onto the GCE, and the change of the current is evaluated to complete the calibration curve. The proposed strategy is capable of cell detection with high sensitivity, selectivity, and simplicity. Besides introducing the electrochemical cell sensing potential of ZC, the present study unravels the cytotoxicity of ZC, which might be attractive and beneficial for a broader scientific community than the sensor community, e.g., to explore ZC in biomedical fields.

## Experiments

### Chemical reagents and apparatus

1-Ethyl-3-(3-dimethyl-aminopropyl) carbodiimide hydrochloride (EDC), and *N-*hydroxysuccinimide (NHS) were purchased from Sigma Aldrich. FeCl_3_⋅6H_2_O, Zn(CH_3_COO)_2_. 2H_2_O, NaOH, l-cysteine, folic acid, and polyethylene glycol were supplied from Merck. All the chemicals were used with analytical grades. The nanoparticle characterization was performed by FESEM-TESCAN MIRA III, XRD-PHILIPS (PW1730), FTIR-Thermo (AVATAR), VSM-LBKFB, and contact angle JIKAN (CGA-20 SE).

### Synthesis of ZnFe_2_O_4_ nanoparticles

In a typical synthesis procedure, 2.195 g of Zn(CH_3_COO)_2_⋅2H_2_O was dissolved in 10 mL of deionized water. Then, 5.406 g of FeCl_3_⋅6H_2_O was added to a beaker containing 10 mL of deionized water and metal salts. The aqueous solutions were kept under stirring for 15 min. These solutions were then mixed to obtain a homogenous solution. Next, the pH was adjusted to 10.5 by the drop-wise addition of NaOH (2 M). The resulting solution was heated up to 120 °C and stirred under reflux for 20 h. The precipitants were then dissolved in abundant water and magnetically collected several times until the pH became neutralized. The final products were obtained through a drying process in an oven at 60 °C for 24 h.

### Functionalization of ZnFe_2_O_4_ nanoparticles with **l**-cysteine, polyethylene glycol, and folic acid (ZC)

The functionalization of ZnFe_2_O_4_ nanoparticles for site-specific targeting was performed with a facile procedure in an aqueous solution. Briefly, 0.6 g of ZnFe_2_O_4_ and 0.25 g of l-cysteine were added to two beakers each containing 50 mL of deionized water and kept under stirring until their dissolution was complete. Then, the l-cysteine solution was poured into the ZnFe_2_O_4_ solution and stirred for 30 min followed by the addition of 0.25 g of PEG. Folate aqueous solutions were prepared through dissolving 150 mg of folic acid in 50 mL of deionized water containing 60 mg of NaHCO_3_. This was done under mild heating followed by the addition of 75 mg of EDC. After 2 h, 150 g of NHS was added to the folate solution, and this mixture was stirred for 15 min. The activated folate solution was then mixed with the ZnFe_2_O_4_ solution, and they were allowed to react overnight at 45 °C. At the end of the reaction, the product was collected with a magnet and dried in an oven at 60 °C overnight.

### Cell culture and the analysis of cell viability

MCF-7 (breast cancer), SKBR-3 (breast cancer), A549 (lung cancer), and Saso (human osteosarcoma) cancer cells were obtained from Pasteur Institute of Iran. The cells were cultivated in 25-cm^2^ flasks containing DMEM (Thermo Fisher Scientific) supplemented with 10% FBS (Thermo Fisher Scientific). The flasks were kept in an incubator at 37 °C. The cells were detached using 0.25% trypsin/EDTA (Thermo Fisher Scientific), and the trypsin activities were inhibited by the addition of a fresh medium. The cells were then collected by centrifugation (rpm = 1300) in a Hettich EBA 20 centrifuge for 5 min. After they were counted with a hemocytometer, 1 mL of a cell suspension in DMEM with various concentrations was prepared in Eppendorf tubes for the cell assay.

The cytotoxicity tests of ZnFe_2_O_4_ and ZC against MCF-7 cells were carried out using the MTT assay according to the established protocol. In brief, the cells with a concentration of 1 × 10^4^ cells/mL were seeded in 96-well plates and allowed to grow for 24 h. Stock solutions were also prepared by the dissolving of 1 mg/mL of nanomaterials in PBS through sonication until a clear orange solution was observed. It was followed by the preparation of the subsequent diluted solutions in a cell culture medium. To conduct MTT (Sigma-Aldrich), the cells were then treated with various concentrations of ZnFe_2_O_4_ and ZC (0, 25, 50, 100, 200, 300, 400, 600, and 800 µg/mL) for 24 and 48 h, respectively. To avoid any possible interference between the nanomaterials and MTT, before the addition of MTT, each well-plate was washed with 200 µL of the medium for three times. Next, 10 µL of MTT with the concentration of 5 mg/mL and 90 µL of DMEM were added to each well followed by incubation for three hours. The medium was then replaced with 150 µL of DMSO, and the well-plates were kept under shaking for more than 15 min to complete the formazan dissolution. Finally, the absorbance was measured with a microplate reader (Stat Fax 2100) at the wave length of 570 nm, and the cell viability was estimated.

### Magnetic cell capture and electrochemical measurements

In this experiment, 100 µL of ZC was added to 1 mL of cell suspension (10^4^ cells/mL) in DMEM followed by shaking for an optimized time. After the cell isolation with a 3 T magnet (20 × 20 × l0 mm^3^), the supernatant was centrifuged, and the cells were suspended in PBS and counted with a hemocytometer. The capture efficiency was calculated according to Eq. (1) in which N_t_ and N stand for the added cells and the founded cells in PBS, respectively.

1 $${\text{Capture efficiency }}\left( \% \right) \, = \, \left[ {\left( {{\text{N}}_{{\text{t}}} - {\text{ N}}} \right)/{\text{N}}_{{\text{t}}} } \right] \times {1}00$$

To perform the electrochemical experiments, the magnetically separated cells were washed with PBS to remove the free cells and then suspended in 50 µL of PBS (pH = 7.4). All the electrochemical measurements were conducted with an Autolab potentiostat/galvanostat (PGSTAT-302 N, Eco Chemie, Netherlands) in three-electrode systems in which a glassy carbon electrode (GCE) was used as a working electrode along with a platinum electrode and Ag/AgCl as counter and reference electrodes, respectively. To prepare the electrode, a GCE (with a 0.2-mm diameter) was polished using 0.3 µm alumina slurry and rinsed with deionized water until a mirror-like surface was obtained. Subsequently, 3 µL of the collected cells was dropped onto the GCE and subjected to electrochemical measurements^40^. All the electrochemical experiments were performed in PBS (pH = 7.4) containing 10 mM [Fe (CN)_6_]^3−/4−^ (Merck) as a redox probe with 0.1 M KCl (Merck)^41,42^. Cyclic voltammetry was conducted in the potential range of − 0.3 to 0.8 V at the scan rate of 100 mV/s. Differential pulse voltammetry (DPV) was carried out from − 0.2 to 0.6 V with the modulation amplitude of 0.025 V at the scan rate of 0.01 V/s. The square wave voltammetry (SWV) was recorded from − 1.2 to 0.8 V with the modulation of 0.05 V and the frequency of 25 Hz. Electrochemical impedance spectroscopy (EIS) measurements were also performed in a frequency range from 0.1 to 100 Hz with the amplitude of 0.05 V.

### Analysis of spiked MCF-7 cells in the blood

The blood samples of healthy volunteers were obtained from Shahid Sadoughi University of Yazd, Iran. MCF-7 cells, as a model of CTCs, were mixed with the same volume of blood samples containing EDTA (Merck). Then, 100 µL of ZC was poured into the blood sample. The ZC-cells (ZC bond to MCF-7 cells) were centrifuged, magnetically collected, washed with PBS, and finally suspended in PBS for further electrochemical cell assay. This study was approved by the Ethics Committee of Yazd University. All the experiments were performed in accordance with the corresponding guidelines, and informed consent was received from all the participants.

## Results and discussion

### Fabrication of a biosensing platform

**Figure 1:**
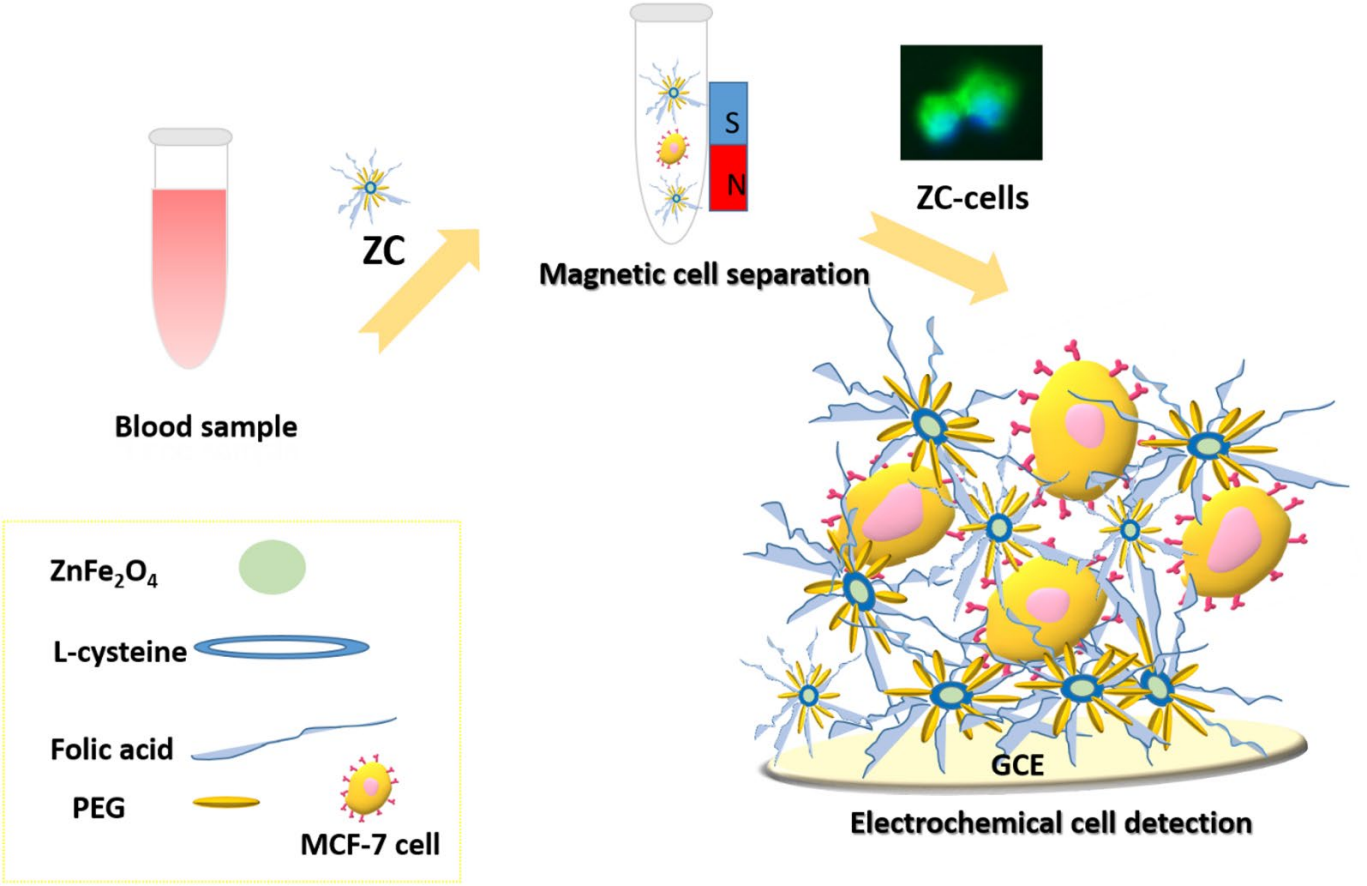
The scheme shows the magnetic separation and the electrochemical detection of MCF-7 using ZC.

The proof-of-principle for the electrochemical monitoring of cancer cells with ZC is schematically depicted in Fig. 1. ZnFe_2_O_4_ nanoparticles were prepared by a simple and eco-friendly hydrothermal method without a calcination process. Zinc acetate as a metal precursor (where acetate functions as a stabilizer) saved the use of any stabilizer/surfactant. To selectively recognize folate-overexpressed cancer cells, folic acid was coupled to l-cysteine via amide bond formation. It is to be noted that folic acid has a high affinity for folate bioreceptors (kd =  ~ 0.1 nM). The cells bonded to ZC were collected with a magnet and then fixed on the surface of a GCE via electrostatic interactions between ZC and the electrode. Magnetic cell separation validates the reliability of cell assay, and the proposed strategy for direct electrochemical cell measurement eliminates the need for chemical agents to modify electrodes.

### Structural characterization of ZnFe_2_O_4_ and ZC

**Figure 2:**
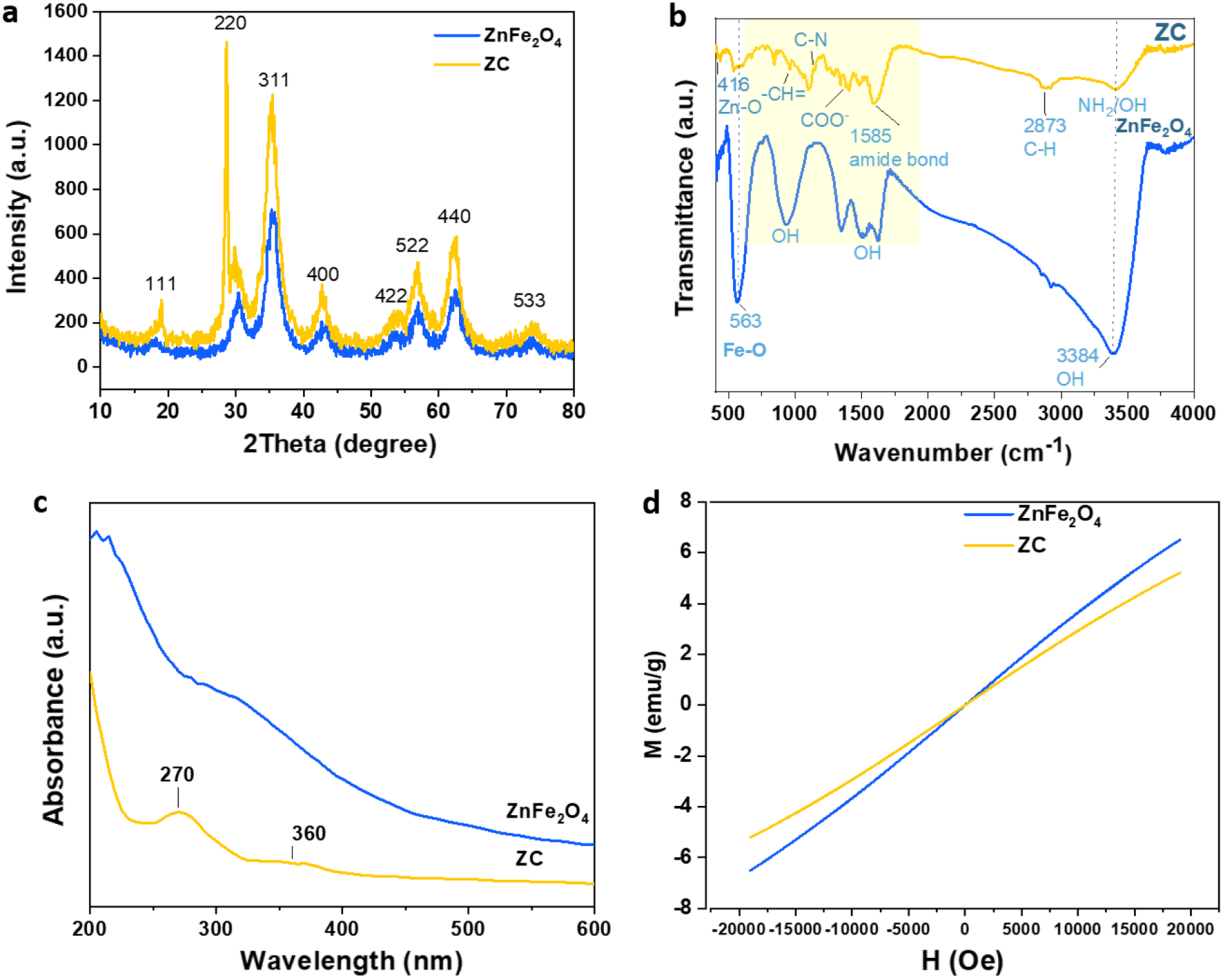
Structural characterization of the synthesized ZnFe_2_O_4_ and ZC: (**a**) XRD pattern, (**b**) FTIR spectrum, (**c**) UV–Vis absorption spectra, and (**d**) magnetization curve of ZnFe_2_O_4_ and ZC.

The crystal structures of ZnFe_2_O_4_ and ZC were studied. The results are shown in Fig. 2a. The diffraction peaks of ZnFe_2_O_4_ and ZC were at the 2Ɵ value of 18.13 (111), 30.18 (220), 35.23(311), 42.9 (400), 53.08 (422), 55.93 (511), 62.43 (440) and 73.43 (533), suggesting that ZnFe_2_O_4_ and ZC involve a cubic spinel phase^13^. The X-ray diffraction (XRD) pattern of ZnFe_2_O_4_ was in agreement with (JCPDS NO. 22-1022)^43,44^. The positions of the diffraction peaks of ZnFe_2_O_4_ and ZC were almost the same, and their high intensities were suggestive of their good crystallinity.

Fourier transform infrared spectroscopy (FTIR) was also performed to verify the chemical structures of ZnFe_2_O_4_ NPs and ZC (Fig. 2b). The spectra of ZnFe_2_O_4_ and ZC show peaks at 416 cm^−1^ and 563 cm^−1^, which can be attributed to the stretching vibration of Zn–O (tetrahedral sites) and Fe–O (octahedral sites). The broad peak at 3384 cm^−1^ in the ZnFe_2_O_4_ spectrum is attributed to the vibration stretching of the O–H groups of the water molecules adsorbed on the ZnFe_2_O_4_ surface during the hydrothermal synthesis^45^. In addition, two vibration peaks at 1500 cm^−1^ and 1612 cm^−1^ are associated with O–H binding, suggesting a large number of hydroxyl groups on the surface of colloidal ZnFe_2_O_4_^46,47^. The ZC exhibits some peaks at 835 cm^−1^ (aromatic CH=), 1191–1281 cm^−1^ (C=C bonds), and 2873 cm^−1^ (C–H groups). The formation of amide bonds between the carboxylic groups in l-cysteine and the amine groups of folic acid was confirmed with a peak at 1585 cm^−1^. The lack of SH bonds in l-cysteine at 2550 cm^−1^ indicates the binding of l-cysteine to ZnFe_2_O_4_ through thiol groups^48^. As the FTIR analysis implied, the C, N, O-containing groups originating from l-cysteine, PEG, and FA could successfully modify the surface of ZC.

The absorption profiles of ZnFe_2_O_4_ and ZC were investigated by UV–Vis spectroscopy to elucidate their optical properties. As Fig. 2c indicates, the Uv–vis spectrum of ZnFe_2_O_4_ contains a peak at 315 nm. Due to the binding of folic acid to ZnFe_2_O_4_, there appeared two main absorption peaks for folic acid at 270 nm and 360 nm in the ZC spectrum. The peaks corresponded to π–π* of aromatic rings and n-π transition, respectively^49^. This confirms that FA was grafted on ZC. The magnetic performances of ZnFe_2_O_4_ and ZC were also explored by the magnetic hysteresis loop technique through applying electric fields (H) in the range of − 20 k to + 20 k at room temperature. According to Fig. 2d, the magnetic saturation (M_s_) of ZnFe_2_O_4_ and ZC were 6.5 and 5.2 emu/g, respectively. The remnant magnetization (Mr) and the coercivity (H_c_) of ZnFe_2_O_4_ and ZC were found to be almost zero. These results are in good agreement with previous studies^13,47^.

**Figure 3:**
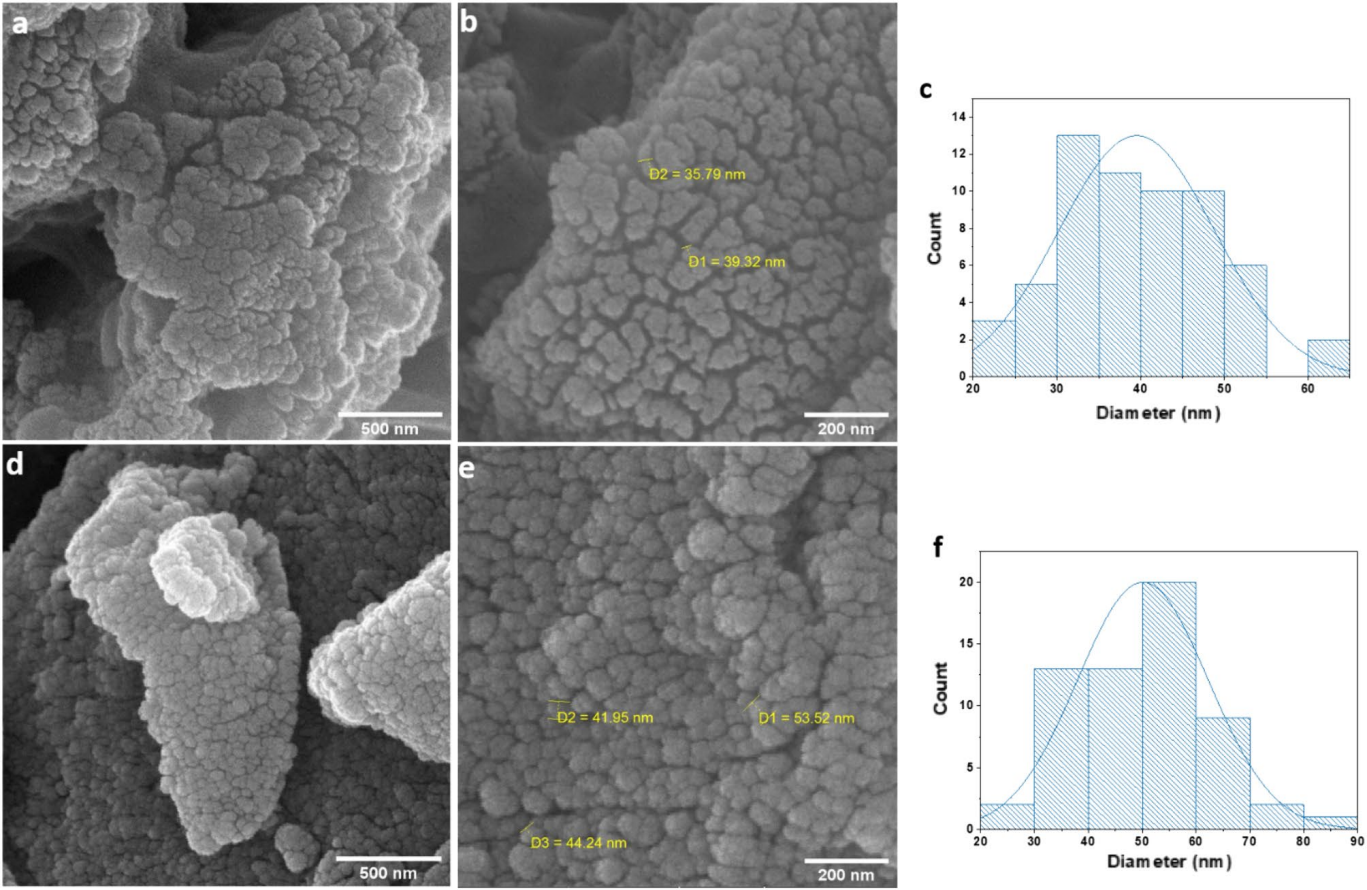
(**a** and **b**) FESEM images of ZnFe_2_O_4_ and (**c**) ZnFe_2_O_4_ particle size distribution and (**d** and **e**) FESEM images of ZC and (**f**) ZC particle size distribution.

**Figure 4:**
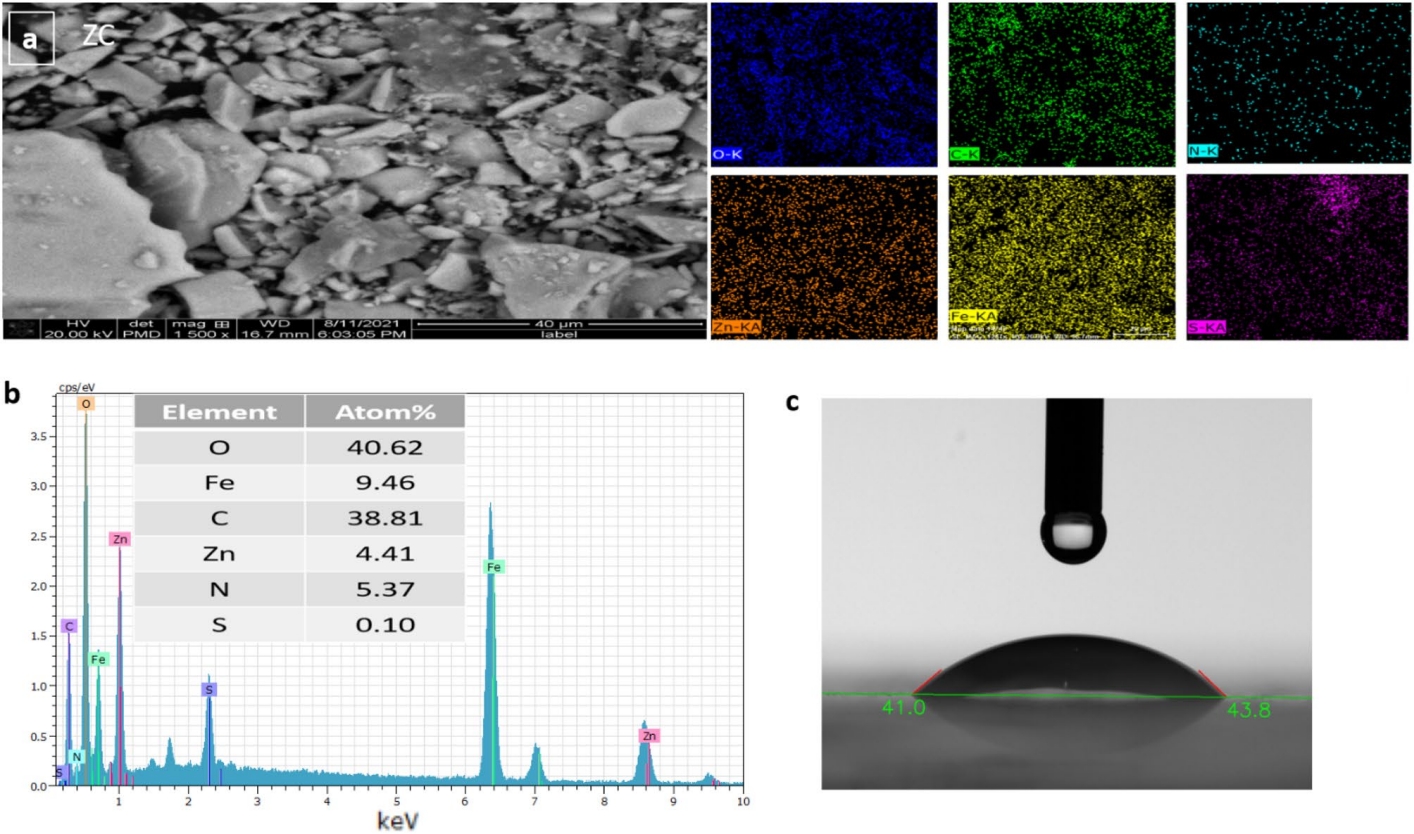
(**a**) Elemental mapping, (**b**) EDS analysis of ZC, and (**c**) contact angel image of ZC.

The surface morphology of ZnFe_2_O_4_ and ZC was studied through field emission scanning electron microscopy (FESEM) (Fig. 4). The diameters of ZC and ZnFe_2_O_4_ were measured with 50 nanoparticles selected in a random manner and by the use of Image J. The FESEM images of ZnFe_2_O_4_ (Fig. 3a,b) show the spherical shape of the nanoparticles. Figure 3c indicates the size distribution of ZnFe_2_O_4_ nanoparticles in the range of 20 to 65 nm. These results are in agreement with a pervious report on the particle size measured by FESEM^47^. Figure 3d,e exhibit the morphology of ZC nanoparticles with different magnifications. As it was found, ZC particles are mainly in irregular spherical shapes. According to Fig. 3f, the size distribution of ZC was in the range of 20 to 90 nm. Moreover, D1, D2, and D3 in the FESEM images show the diameters of some nanoparticles. The histograms in Fig. 3c,f were fitted to normal distributions, and then the average particle sizes of 39 and 50 nm were found for ZnFe_2_O_4_ and ZC, respectively..

Figure 4a shows the elemental mapping of ZC, which proves the existence of homogenously distributed oxygen, carbon, iron, zinc, sulfur, and nitrogen in ZC. The chemical composition of ZC was also determined through energy dispersive spectroscopy (EDS) (Fig. 4b). In this regard, the elemental contents and their atomic percentages included O (40.62%), Fe (9.46%), Zn (4.41%), C (38.81%), N (5.37%), and S (0.10%). The high percentage of carbon further proves the functionalization of ZnFe_2_O_4_.

To improve fouling resistance, one established strategy is the preparation of a highly hydrophilic surface using a hydrophilic polymer such as PEG. Enhanced hydrophilicity prevents the adsorption of proteins or non-specific molecules onto nanomaterials. In this study, the average contact angle of ZC was 44.5°, which indicates satisfying hydrophilicity (Fig. 4c). This low contact angle suggests impeding the absorption of contaminants on the surface of ZC due to the formation of a hydrophilic layer.

### Cytotoxicity and magnetic cell capture

**Figure 5:**
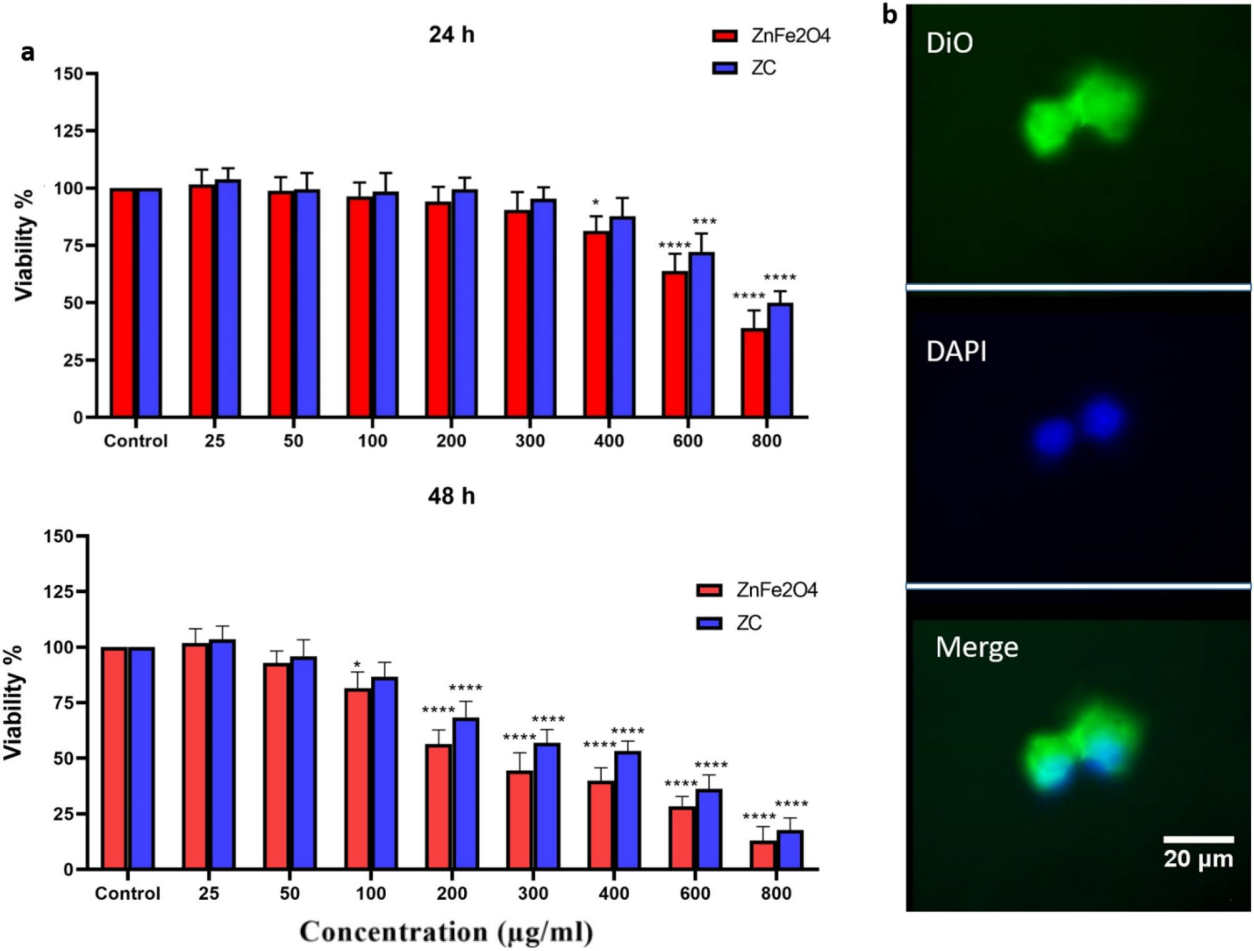
(**a**) MTT assay of ZnFe_2_O_4_ and ZC against MCF-7 cells after 24 and 48 h, (**b**) Fluorescence images of MCF-7 cells: The ZC labeled with DiO (green) and the DAPI (blue) indicates the nucleus. The data points represent the mean ± sd of triplicates. *p < 0.05, ***p < 0.001, and ****p ≤ 0.0001 were considered statically significant according to a paired t-test.

The in vitro cytotoxicity examination of ZnFe_2_O_4_ and ZC with various concentrations from 0 to 800 µg/mL against MCF-7 cells was performed using the standard MTT assay. As shown in Fig. 5a, ZnFe_2_O_4_ and ZC had negligible cytotoxicity effect, compared to the control groups in concentrations up to 400 µg/mL after 24 h of incubation.

When the cells were exposed to the prepared nanomaterials for longer time (48 h), there was negligible toxicity in concentrations up to 100 µg/mL. However, with an increase in the concentration of the nanomaterials, the cell viability reduced. It is concluded that the cytotoxicity of ZnFe_2_O_4_ and ZC depends not only on their dose but also on the exposure time. Due to its low toxicity even after 48 h of treatment, ZC has potential to be employed for capturing patient-derived cancer cells and re-culturing those captured cells for further analysis (e.g., genome analysis, tumor mapping, drug side effect).

To validate the ability of ZC to recognize MCF-7 cells, ZC was labeled with DiO (green), as a fluorescent probe, and its nucleus was stained with DAPI (blue). The obtained fluorescence images are shown in Fig. 5b. The ZC was found tightly bound to the MCF-7 cell membranes.

To determine the optimal time for the cell capturing, ZC was kept under shaking with cancer cells (10^4^ cells/mL) for various times (10, 20, 30, 40, 60, 80, 100 min) and then separated with a magnet. The results are presented in the supplemented file (S1). Accordingly, the optimum incubation time was determined to be 60 min. A cell capture efficiency of 83–88% was also observed for different cell concentrations (25,150, 500, 1000, 10,000 cells/mL). Our system has lower capture efficiency than a pervious microfluidic device (99.5% at 0.6 ml/h), but it is simpler and cheaper^50^. Over recent years, microfluidic chips have gained considerable attention for cell isolation^51,52^. In microfluidics, capture efficiency and cell purity can often be achieved more efficiently than with nanomaterials; however, bubble formation or cell entrapment can restrict their clinical use.

### Characterization of the ZC-based biosensing platform

**Figure 6:**
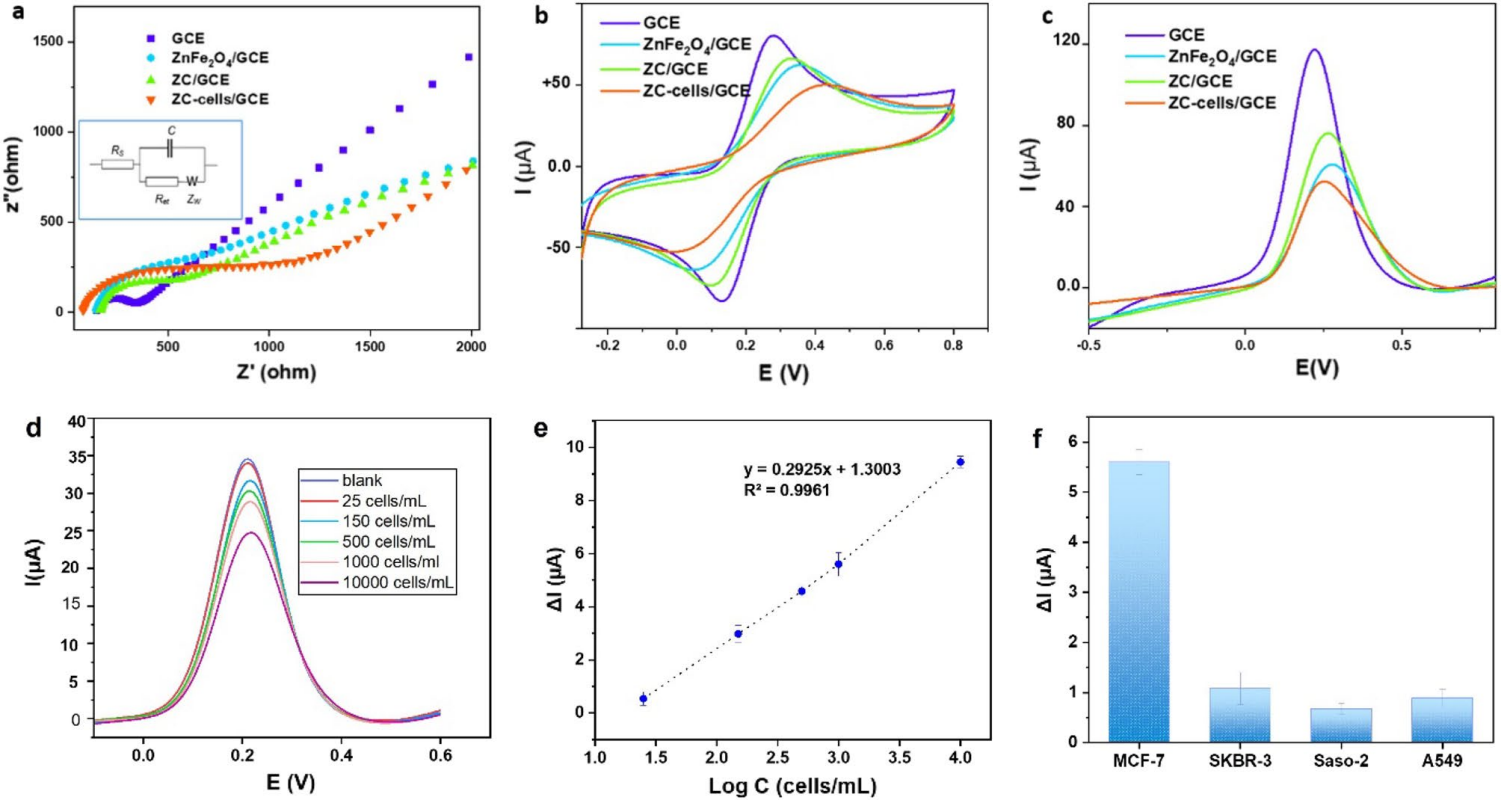
(**a**) EIS, (**b**) CV and (**c**) SWV responses of the bare GCE, ZnFe_2_O_4_/GCE, ZC/GCE, and ZC-cells/GCE, (**d**) DPV response of different concentrations of MCF-7, (**e**) regression analysis of ΔI toward the logarithmic concentration of MCF-7 cells (ΔI represents the current decrease of the blank versus different concentrations of MCF-7 cells, (**f**) the selectivity of the cytosensor, (inset a) the schematic display of Randle's equivalent circuit (error bars represent the standard deviations, n = 3).

The electrochemical performance of the biosensing platform in the presence of ZnFe_2_O_4_, ZC, and ZC bond to MCF-7 cells (ZC-cells) was characterized using three techniques including EIS, CV, and SWV. The results of the characterization are provided in Fig. 6a–c. The EIS technique could sensitively and rapidly indicate the change in the electron resistance of the redox probe, [Fe (CN)_6_]^3−/4−^, at the electrode interfaces. An EIS diagram, as presented in Fig. 6a, consists of a semicircle to show a high frequency and a linear part at a low frequency. The blocking of the electron transport on the electrode affects the diameter of the semicircle, which is suggestive of electron resistance (R_et_)^53^. The linear part corresponds to the diffusion process on the electrolyte–electrode interface^54^. The R_et_ values were estimated according to the equivalent circuit of the Nyquist curve presented in the inset of Fig. 6a. The R_et_ of the GCE was found to be 371 Ω, suggesting the rapid electron transfer. The Nyquist curves of ZnFe_2_O_4_ and ZC had the R_et_ values of 646 Ω and 406 Ω, respectively. Placing the captured cells on the electrode changed the electrochemical properties of the electrode including its R_et_. As expected, once the ZC cells were put on the GCE, the diameter of the semicircle enlarged (R_et_ = 793 Ω), which indicated the sensitive electrochemical response of the biosensing platform to captured cells. The EIS results showed that the cell monitoring using ZC was successful. Figure 6b depicts the CV responses from the redox reaction of [Fe (CN)_6_]^3−/4−^ at the GCE, ZnFe_2_O_4_/GCE, ZC/GCE, and ZC-cells/GCE. As it can be seen, the modification with ZnFe_2_O_4_ and ZC made the anodic peak potential of [Fe (CN)_6_]^3−/4^ on the GCE shift from 0.276 V to 0.354 and 0.334 V, respectively. The CV responses (i.e., the values of the current and the peak-to-peak potential separation) suggest that the electrochemical performance of ZC was slightly better than that of ZnFe_2_O_4_. It is probably due to the redox activity associated with the SH group of l-cysteine, which promoted the electron transfer at the electrode surface and increased the current value^55^.

With the addition of ZC cells to the GCE, the anodic peak potential of the redox probe had a dramatic shift to 0.420 V, proving that the magnetic cell isolation blocked the access of the redox probe to the electrode surface. Further decrease in the anodic currents of the redox probe in the presence of ZC-cells, in comparison with ZC, originated from the formation of an insulating layer on the electrode surface to inhibit the electron transfer^13^. The interactions between ZC and ZnFe_2_O_4_ with [Fe (CN)_6_]^3−/4^ need to be further studied beyond the scope of this research. Figure 6c shows the SWV currents for the GCE, ZnFe_2_O_4_/GCE, and ZC-cells/GCE. Based on the SWV results, after the modification of the GCE with ZnFe_2_O_4_ and ZC, the current was decreased from 120 µA to 70 µA and 106 µA, respectively. The SWV technique also proved the lower conductivity of ZnFe_2_O_4_ than ZC. The capturing of the cells by ZC led to further reduction in the current (59 µA). The SWV results were consistent with the EIS and CV responses.

### Performance of the ZC-based biosensing platform

Encouraged by the high sensitivity of the electrochemical platforms, the magnetically captured cells were transferred to the GCE for quantification with DPV currents. As Fig. 6d shows, the decrease in the value of the current response was due to the number of the cells captured with ZC. The calibration curve of ΔI (I_b_ − I_c_, where I_b_ stands for the blank current, ZC, and I_c_ refers to the current of ZC-cells) and the logarithm of the cell concentration was obtained in a range from 25 to 10^4^ cells/mL (Fig. 6e). The detection limit of the platform was estimated to be 3 cells/mL (S/N = 3). Generally, the sensitivity of cell detection depends on the binding affinity between tumor bioreceptors and biorecognition elements, the ability to translate the recognition events into measurable signals, and anti-interference properties. The excellent cytosensing performance of the proposed biosensing platform can be the result of the properly selected materials and the cell enrichment on the electrode through out-suite magnetic cell trapping by ZC, which made a strong bond to the recognition sites on the cells and improved the detection sensitivity. Recently, a ratiometric cytosensor has been established with Fe_3_O_4_@SiO_2_@Au nanoparticles coupled to a DNA walker and a surface-enhanced Raman spectroscopy tag (Au nanoparticles labeled with DNA and Raman molecules), resulting in a detection limit as low as one cell^56^. Compared to the previous reports ^40,57–59^, our biosensing platform has the great advantage of being developed with no need for a complex instrument, time-consuming multiple steps, or labeling while offering remarkable sensitivity.

The selectivity of the biosensing platform was analyzed by the DPV current of MCF-7 cells (target cells), and low/negative-level folate expressed cells including SKBR-3, A549, and Saso-2. As Fig. 6f shows, the current response to MCF-7 cells is remarkable as compared to the non-target cells. To evaluate the reproducibility of the proposed biosensing platform, five sensors were prepared independently and employed to measure MCF-7 cells with a concentration of 10^3^ cells/mL. The relative standard deviation (RSD) was estimated to be approximately 5.08%, demonstrating acceptable reproducibility.

The feasibility of the biosensing platform for the detection of folate-enriched cancer cells in clinical samples was evaluated through a spiked experiment. MCF-7 cells (150, 500, and 1000 cells/mL) were spiked in 1 mL of the lysed blood samples of healthy volunteers. The recovery rates were 103.76%, 96.02% and 100.20%, respectively. PEG's antifouling property, out-suite magnetic cell enrichment from the blood sample and FA's high affinity for folate bioreceptors are considered to be the key factors responsible for the high performance of the presented biosensing platform.

## Conclusion

In summary, affinity-based ZnFe_2_O_4_ nanomaterials were synthesized in an aqueous solution and used for magnetic cell isolation by means of an external magnetic field. The application of ZnFe_2_O_4_ for cancer cell detection and isolation was reported here for the first time. To evaluate the research method, MCF-7, found in the peripheral blood of breast cancer patients, was chosen as a model of CTCs. As an MTT assay revealed, the cytotoxicity of ZC and ZnFe_2_O_4_ is dose- and time-dependent. The high biocompatibility of ZC might inspire researchers to explore ZC in various fields such as targeted drug delivery, tissue engineering, magnetic resonance imaging (MRI), magnetic hyperthermia and wound healing. Visualized staining experiments by fluorescence images verified the targeting capability of ZC for the interaction with MCF-7 cells. Under optimum conditions, MCF-7 cells were magnetically captured and transferred to a GCE for quantification. The spiked experiments demonstrated the ability of the ZC-based biosensing platform in blood samples. The proposed strategy is easy to operate and can be adopted for other circulating tumor markers, such as exosomes, through proper targeting ligands.

## Supplementary Information

Supplementary Figures.

## Data Availability

The datasets used and/or analyzed during the current study are available from the corresponding author on a reasonable request.
