# Supplementary material for: Facile preparation of a cost-effective platform based on ZnFe2O4 nanomaterials for electrochemical cell detection
Source: Sci Rep. 2023 Mar 27;13:4962. doi: 10.1038/s41598-023-31377-2 (PMC10042879; doi:10.1038/s41598-023-31377-2)
Supplement: Supplementary file 1 — Supplementary Figures. [file 41598_2023_31377_MOESM1_ESM.docx]

**Supplementary Information for:**

**Facile preparation of a cost-effective platform based on ZnFe_2_O_4_ nanomaterials for electrochemical cell detection**

Fereshteh Vajhadin^1^, Mohammad Mazloum-Ardakani^*,1^, Mahdie Hemati ^2,3^, Seyed Mohammad Moshtaghioun^4^

^1^ Department of Chemistry, Faculty of Science, Yazd University, Yazd, 8915818411, Iran

^2^ Department of Clinical Biochemistry, Faculty of Medicine, Shahid Sadoughi University of Medical Sciences, Yazd, Iran

^3^Medical Nanotechnology & Tissue Engineering Research Center, Yazd Reproductive Sciences Institute, Shahid Sadoughi University of Medical Sciences, Yazd, Iran

^4^Department of Biology, Faculty of Science, Yazd University, Yazd, Iran

E-mail: [mazloum@yazd.ac.ir](mailto:mazloum@yazd.ac.ir) (M.Mazloum-Ardakani)

**List of supporting information:**

S1. Magnetic cell separation

S2. CV curves of ZnFe_2_O_4_/GCE, ZC/GCE, and GCE in buffer solution

S1. Magnetic cell separation


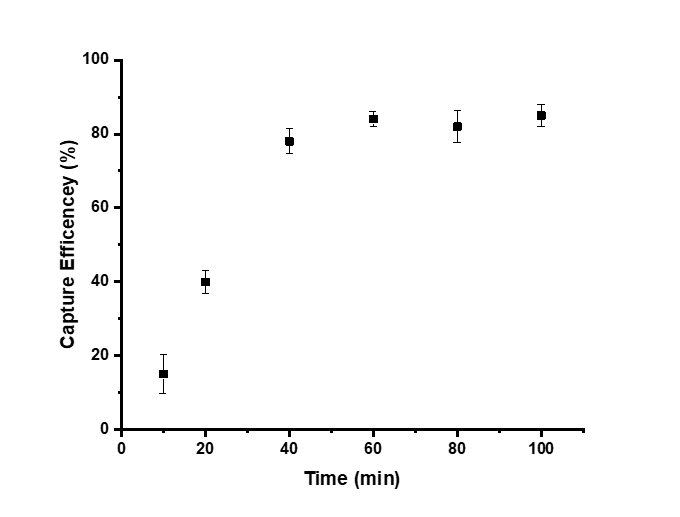


S1. Cell capture efficiency with ZC at various incubation times (n=3).
